# Vital Statistics

**Published:** 1915-11

**Authors:** 


**Vital Statistics.** Frederick L. Hoffmann, LL.D., Statistician of Prudential Life Ins. Co., Pac. Med. Jour. Aug. Approximate Density of the World's Population estimated by continents for the year 1911.

| Continent | Area in Square Miles | Population | Population per Sq. Mile |
|---|---|---|---|
| Europe | 3,833,567 | 463,997,000 | 121.0 |
| Asia | 16,997,639 | 962,233,000 | 56.6 |
| Africa | 11,760,689 | 135,987,000 | 11.6 |
| North America | 8,631,657 | 127,993,000 | 14.8 |
| South America | 7,184,021 | 51,191,000 | 7.1 |
| Australasia | 3,317,762 | 7,572,000 | 2.3 |
| Total land area | 51,725,335 | 1,748,975,000 | 33.8 |

Does not include the practically uninhabited polar regions.

Considered by local areas in which cholera was particularly virulent, it appears that there were towns in which the death rate attained to the almost inconceivable proportion of 97.35 per one thousand (Gaya), of which 11.87 was caused by cholera, 35.61 by fevers, and 19.99 by plague. Such conditions are extremely rare in modern civilized communities, although as illustrated in the cholera epidemic of the city of Hamburg, the menace of serious local outbreaks is by no means a remote possibility. The sanitary security of modern countries depends largely upon the highest attainable degree of efficiency in the control of so-called international diseases, and in this respect no country in the world has a better public health service than the United States.

Population Density. The effect of excessive death rates on population ingrease is so obvious as not to require extended consideration. India in 1911 had a birth rate of 38.6 per one thousand, and a death rate of 32. But for the prevalence of epidemic and largely preventable diseases the natural increase in population would have been much greater than was actually the case.

The lowest death-rate is 10.7: 1000 population (which cannot be regarded as possible except by immigration of healthy adults, In a stationary population, such a mortality would imply an average life of nearly 100 years. In a normal population, with a high birth rate, such a mortality would be possible on a considerably lower longevity average, on account of the dilution of the population from below, were it not for the inevitably high death rate of infants). On the other hand, Spain has a mortality of 23.7, Hungary 25, Russia 30.5, Mexico 31.1, India 33.2 and 20 cities of Egypt 40.9. (Probably a century ago, 30 was not far from the "normal" death rate of most civilized, fairly well populated countries of temperate climates).

Comparing the present with 30 years ago, the most marked decline of the death rate is in the Netherlands, to 63% of the former. A similar comparison shows 76% for the U. S., 71% for England and Wales, Denmark, Australia and Italy, 72% for Belgium and Germany, 67% for Switzerland.

General Death Rate of the United States Registration Area, 1880-1913:

| | | | |
|---|---|---|---|
| 1880 | 8,538,000 | 169,060 | 19.8 |
| 1890 | 19,659,618 | 386,939 | 19.6 |
| 1900 | 30,765,618 | 539,939 | 17.6 |
| 1905 | 34,094,605 | 545,533 | 16.0 |
| 1910 | 53,843,896 | 805,412 | 15.0 |
| 1911 | 59,275,977 | 839,284 | 14.2 |
| 1912 | 60,427,133 | 838,251 | 13.9 |
| 1913 | 63,299,164 | 890,823 | 14.1 |

Decline in the Death Rate of Cities. During the last thirty years the death rate of large cities of this and other countries has declined as follows, the comparison being limited to the quinquennial periods ending respectively with 1885 and 1910: The death rate of London decreased from 20.9 to 14.0 per one thousand; of Dublin, from 27.5 to 21.6; of Paris, from 24.4 to 17.5; of Amsterdam, from 25.1 to 13.1; of St. Petersburg, from 32.8 to 25.5; of Berlin, from 26.5 to 15.5; of Vienna, from 28.2 to 17.0; of Budapest, from 31.5 to 19.5; of Milan, from 30.3 to 19.3; of Melbourne, Victoria, from 20.1 to 13.1; of Sydney, New South Wales, from 20.8 to 10.5; of New York, from 27.5 to 17.0; of Chicago, from 21.5 to 14.5; and of Philadelphia from 22.3 to 17.7.

It has been very generally held by sociologists that wars, pestilence and famine, have had a conservative action in preventing overcrowding. The natural increase in population may exceed 1% per annum (the increase in the U. S. of about 20% per decade is at least half due to immigration). At 1% increase per annum, money or population will double in a trifle less than 70 years. Thus, in 140 years, the average density of the whole habitable world would be slightly greater than that of Europe. While, of course, many areas, even whole countries, enjoy prosperity with much greater density of population, they do so in the same way as cities, by exchanging manufactures for food, or, as in the case of Belgium, by specializing on certain crops not sufficient in themselves to support the population. It would require about 300 years for the population of the world to increase 10 times, to bring it to a density of about that of Germany at present, which has appar ently been shown to be just about self-supporting under present agricultural and economic conditions.

